# Molecular Characterization and Expression Profiling of Odorant-Binding Proteins in *Apolygus lucorum*

**DOI:** 10.1371/journal.pone.0140562

**Published:** 2015-10-14

**Authors:** Hai-Bin Yuan, Yu-Xiao Ding, Shao-Hua Gu, Liang Sun, Xiao-Qiang Zhu, Hang-Wei Liu, Khalid Hussain Dhiloo, Yong-Jun Zhang, Yu-Yuan Guo

**Affiliations:** 1 College of Agronomy, Jilin Agricultural University, Changchun, Jilin, 130118, China; 2 State Key Laboratory for Biology of Plant Diseases and Insect Pests, Institute of Plant Protection, Chinese Academy of Agricultural Sciences, Beijing, 100193, China; 3 Key Laboratory of Tea Plants Biology and Resources Utilization of Agriculture Ministry, Tea Research Institute, Chinese Academy of Agricultural Sciences, Hangzhou, 310008, China; 4 Department of Entomology, Faculty of Crop Protection, Sindh Agriculture University Tandojam, Pakistan; INRA-UPMC, FRANCE

## Abstract

*Apolygus lucorum* (Meyer-Dür) (Hemiptera: Miridae) is one of the most important agricultural pests, with broad host range and cryptic feeding habits in China. Chemosensory behavior plays an important role in many crucial stages in the life of *A*. *lucorum*, such as the detection of sex pheromone cues during mate pursuit and fragrant odorants during flowering host plant localization. Odorant-binding proteins (OBPs) are involved in the initial biochemical recognition steps in semiochemical perception. In the present study, a transcriptomics-based approach was used to identify potential OBPs in *A*. *lucorum*. In total, 38 putative OBP genes were identified, corresponding to 26 ‘classic’ OBPs and 12 ‘Plus-C’ OBPs. Phylogenetic analysis revealed that *A*. *lucorum* OBP proteins are more closely related to the OBP proteins of other mirid bugs as the same family OBP clustering together. Quantitative real-time PCR analysis for the first reported 23 AlucOBPs revealed that the expression level of 11 AlucOBP genes were significantly higher in antennae of both sexes than in other tissues. Three of them were male antennae-biased and six were female antennae-biased, suggesting their putative roles in the detection of female sex pheromones and host plant volatiles. In addition, three, four, two and one AlucOBPs had the highest degree of enrichment in the stylet, head, leg, and in abdomen tissues, respectively. Two other OBPs were ubiquitously expressed in the main tissues, including antennae, stylets, heads, legs and wings. Most orthologs had similar expression patterns, strongly indicating that these genes have the same function in olfaction and gustation.

## Introduction

The mirid bug *Apolygus lucorum* (Meyer-Dür) (Hemiptera: Miridae), is a dominant pest in northern China [1]. During the past decade, due to widespread planting of Bt cotton and an associated drop in the use of broad-spectrum insecticides, *A*. *lucorum* has become a serious pest of cotton and many other crops [2, 3]. *A*. *lucorum* is a polyphagous pest with a wide range of host plants including many arable crops, vegetables, stone fruits, ornamentals, and pasture plants [4]. Currently, the application of chemical insecticides is the primary strategy to control *A*. *lucorum*. However, the intensive use of insecticides leads to problems like pest resistance, pest resurgence and insecticide residues [5]. Hence, environmentally friendly, cost-effective and sustainable management tactics should be employed for the effective control of *A*. *lucorum*.

During the long-term co-evolution of plants and insects, a sensitive and specific olfactory system has contributed to the perception of semiochemicals in the environment. Insect olfactory perception has been implicated in a wide range of activities, including the detection of food sources, egg-laying substrates, mating and predating [6]. Insect antennae, the most significant sensory organs of olfaction, typically contain thousands of sensilla and associated sensory neurons. Studies on the internal structure of different types of sensilla of moths and mirid bugs suggested that sensilla trichodea and sensilla basiconica with multiple pores and neurons might have olfactory functions, while sensilla chaetica without pores and neurons might function in mechanical perception [7–9]. In addition, single sensillum recordings indicates that sensilla trichodea house olfactory receptor neurons responding to sex pheromones, volatiles and volatile derivatives [10–12].

In insects, a variety of odor-related proteins, such as odorant-binding proteins (OBPs), chemosensory proteins (CSPs), odorant receptors (ORs), sensory neuron membrane proteins (SNMPs) and odorant degrading enzymes (ODEs), complete olfactory perception. Insect OBPs belong to a family of relatively small (130–150 amino acids, 15–17 kDa), water-soluble, globular proteins in extremely high concentrations (up to 10 mM) in the antennal sensillar lymph [13, 14]. It has been suggested that OBPs might act as molecular carriers that solubilize and transport fat-soluble odorants, which have access to the sensillar lymph via cuticular pores, across the sensillar lymph to the olfactory receptors in the olfactory neuronal membrane [13, 15, 16]. In addition, OBPs might also participate in other physiological functions, such as detoxification [17], and the discrimination of taste compounds [18–21]. Most OBPs have six highly conserved Cys residues, paired in three interlocked disulfide bridges, although there is low sequence similarity among the OBPs in different insects species [22]. OBPs can be categorized into ‘classic’ OBPs (six Cys residues), ‘Plus-C’ OBPs (a Pro residue and two additional Cys residues), ‘Minus-C’ OBPs (lacking two of the six conserved Cys), ‘atypical’ OBPs (9–10 Cys residues) based on the number of conserved Cys residues and the molecular structure of the OBPs [22].

Vogt [15] identified the first insect OBP from *Antheraea polyphemus* male antennae. Studies have demonstrated that insect OBPs are highly expressed in antennae, which are associated with olfactory perception. In Lepidoptera, such as *Helicoverpa armigera* [23], and *Sesamia inferens* [24], OBPs showed antennae-specific expression. In Hemipteran, such as *Lygus lineolaris*, *Adelphocoris lineolatus*, *Aphis gossypii*, *Nilaparvata lugens* and *Sogatella furcifera*, a large proportion of identified OBP genes are highly expressed in antennae [25–29]. Our previous study has identified 12 OBPs in *A*. *lucorum*, and among those, five OBPs were highly expressed in the antennae [30]. Immunohistochemical labeling and fluorescence in situ hybridization (FISH) have further defined the location of OBPs in different types of antennal sensilla. In Lepidoptera, long sensilla trichodea primarily express pheromone-binding proteins (PBPs), which are involved in the perception of sex pheromones released from female moths [31, 32], while short sensilla trichodea and sensilla basiconica primarily express general odorant-binding proteins (GOBPs), which are involved in the perception of the host plant volatiles [33, 34]. In Hemipteran, OBPs are expressed in different types of sensilla, such as AlucOBP7 was located on sensilla trichodea and sensilla basiconica [35], and AlinOBP1 was also located on sensilla trichodea and sensilla basiconica [36], but AlinOBP13 was only located on short sensilla basiconica [37]. Remarkably, subsequent studies have identified OBPs that are highly expressed not only in the antennae, but also in other tissues, such as the legs and heads. Many scholars have suggested that the OBPs that are highly expressed in these tissues might be associated with taste perception and might participate in other physiological functions [19, 21].

The diverse expression profiles of insect OBPs make it possible for these proteins to participate in different physiological functions. At present, fluorescence competitive binding assays have been widely used to examine the binding capacity of recombinantly expressed OBPs for specific odorant ligands to assess the role of OBPs in olfaction. In Hemipteran, AlinOBP1 exhibited high binding abilities with two major putative pheromone components, ethyl butyrate and trans-2-hexenyl butyrate and six cotton volatiles, namely, octanal, nonanal, decanal, 2-ethyl-1-hexanol, β-caryo-phyllene and β-ionone [36]. AlinOBP13 showed a more specific binding preference to terpenoids [37]. Due to the limited number of ligands tested, AlucOBP7 exhibited binding affinities with plant volatiles, methylis salicylate and pheromone analogs, such as 4-oxo-(*E*)-2-hexenal [35].

While the molecular basis of insect olfaction has been extensively examined in holometabolous insects (e.g., Lepidopterans and Dipterans), the molecular components and mechanisms comprising the Hemipteran olfactory system have not been fully elucidated. In field experiments, the main sex pheromone components, 4-oxo-(*E*)-2-hexenal and trans-2-hexenyl butyrate, could attract the adult males of *A*. *lucorum* [38]. In addition, *A*. *lucorum* has a preference for flowering host plants [39]. Using coupled gas chromatography-electroantennography (GC-EAD) and gas chromatography-mass spectrometry (GC-MS), the volatiles of three preferred host plants (*Artemisia argyi* Lévl. et Vant., *Artemisia lavandulaefolia* DC. and *Artemisia annua* L.) including (*Z*)-3-hexen-1-ol, m-xylene, butyl acrylate, butyl propionate, butyl butyrate and (*Z*)-3-hexenyl acetate were detected [40]. However, *A*. *lucorum* OBPs that could effectively bind sex pheromone components and/or aromatic plant volatiles have not been identified.

Till now, our understandings for the molecular components comprising the *A*. *lucorum* system is even more incomplete with sequence and expression data currently limited to 15 identified *A*. *lucorum* OBP genes [20, 30]. To elucidate the molecular basis for olfactory reception of *A*. *lucorum* and to facilitate the design and implementation of novel intervention strategies against these plant bugs [41], we used an antennal transcriptomics screening approach to identify OBP genes, and subsequently examined OBP gene expression in all body tissues by using quantitative real-time PCR (qRT-PCR).

## Methods and Materials

### Ethics Statement

*A*. *lucorum* is a common agricultural insect pest and is not included in the “List of Endangered and Protected Animals in China”. All operations were performed according to ethical guidelines in order to minimize pain and discomfort to the insects.

### Insect material and RNA extraction

*A*. *lucorum* nymphs and adults were collected from cotton fields at the Langfang Experimental Station of the Chinese Academy of Agricultural Sciences (CAAS), Hebei Province (39.53°N, 116.70°E), China. Because both Langfang Experimental Station and Institute of Plant Protection belong to Chinese Academy of Agricultural Sciences (CAAS), therefore we didn't need any specific permission to collect insect materials form this area. The *A*. *lucorum* colony was fed with fresh corn and maintained at 29 ± 1°C, 60 ± 5% relative humidity (RH), and 14:10 light: dark (L:D) in the laboratory. For transcriptome sequencing, antennae (500 each sex) were collected from 4-days-old male and female adult individuals. For qRT-PCR, different tissues were collected in three batches, each batch included 500 male antennae, 500 female antennae, 1000 male stylets, 1000 female stylets, 100 male heads without antennae and stylets, 100 female heads without antennae and stylets, 50 male thoraxes, 50 female thoraxes, 50 male abdomens, 50 female abdomens, 100 male legs, 100 female legs, 100 male wings and 100 female wings. All collected tissues were immediately frozen in liquid nitrogen, and stored at -80°C until further processing. Total RNA was extracted from the antennae and other tissues using Trizol reagent (Invitrogen, Carlsbad, CA, USA) according to the manufacturer’s instructions. The quantity of RNA samples was assessed using 1.1% agarose gel electrophoresis and a NanoDrop 2000 spectrophotometer (NanoDrop, Wilmington, DE, USA).

### cDNA library construction, Illumina sequencing

The integrity of total RNA was assessed using the RNA Nano 6000 Assay Kit of the Agilent Bioanalyzer 2100 system (Agilent Technologies, CA, USA). A total of 3 μg of RNA from male and female antennae, respectively, was used as input material for the RNA sample preparations. Briefly, mRNA was purified from total RNA using poly-T oligo-attached magnetic beads. Fragmentation was accomplished using divalent cations under elevated temperature in NEBNext. First-Strand Synthesis Reaction Buffer (5×). First-strand cDNA was synthesized using random hexamer primer and M-MuLV Reverse Transcriptase (RNaseH). Second-strand cDNA synthesis was subsequently performed using DNA Polymerase I and RNaseH. The remaining overhangs were converted into blunt ends via exonuclease RNase/polymerase activities. After adenylation of the 3' ends of the DNA fragments, NEBNext Adaptor with hairpin loop structures were ligated for hybridization. To select cDNA fragments of preferentially 150–200 bp in length, the library fragments were purified using the AMPure XP system (Beckman Coulter, Beverly, USA). Subsequently, 3 μl of USER Enzyme (NEB, USA) was used with size-selected, adaptor-ligated cDNA at 37°C for 15 min followed by 5 min at 95°C. Next, PCR was performed using the Phusion High-Fidelity DNA polymerase, Universal PCR primers and Index (X) Primer. The PCR products were purified (AMPure XP system), and the library quality was assessed using an Agilent Bioanalyzer 2100 system. The clustering of the index-coded samples was performed on a cBot Cluster Generation System using a TruSeq PE Cluster Kit v3-cBot-HS (Illumina) according to the manufacturer’s instructions. After cluster generation, the library preparations were sequenced on an Illumina Hiseq 2500 platform and paired-end reads were generated. *De novo* assembly was performed with the short reads assembly program Trinity [42].

### Screening putative OBP transcripts in the *A*. *lucorum* antennal transcriptome

The tBLASTn program was performed, using known OBP protein sequences of Hemiptera as the “query”, to screen the transcriptome database for identifying candidate unigenes that encode putative OBP genes. All putative OBP genes were manually confirmed using the BLASTx program against the NR nucleotide database of the National Center for Biotechnology Information (NCBI) with a cut-off E-value = 1.0E-5.

### Gene cloning of *A*. *lucorum* OBPs

Five low abundant OBPs transcripts that were not found in the *A*. *lucorum* antennal transcriptome were obtained through gene cloning. Specific primers were designed by referring to the previously identified AlucOBP genes [20, 30] (S1 Table).

### Verification of OBP genes by cloning and sequencing

All the AlucOBP nucleotide sequences obtained from the *A*. *lucorum* antennal transcriptome were confirmed by gene cloning and sequencing. The full-length AlucOBP genes were amplified with ExTaq DNA polymerase (TaKaRa, Dalian, Liaoning, China) by PCR using gene-specific primers (S2 Table). The PCR amplification conditions were set as 94°C for 4 min, followed by 35 cycles of 94°C for 30 s, 55°C for 30 s, and 72°C for 40 s, and a final 10 minutes of elongation step at 72°C. After analysis on 1.5% agarose gel, the PCR products were sub-cloned into the pGEM-T Easy vector for sequencing.

### Comparative analysis of OBP transcripts between sexes

To compare the differential expression of OBP genes in the *A*. *lucorum* male and female antennal transcriptomes, the read number for each OBP gene between male and female antennae was converted to RPKM (Reads Per Kilobase per Million mapped reads) [43], using the formula: RPKM (A) = (1,000,000 × C × 1,000) / (N × L), where RPKM (A) is the expression of OBP gene A, C is the number of reads uniquely aligned to OBP gene A, N is the total number of reads uniquely aligned to all unigenes, and L is the number of bases in OBP gene A. The RPKM method eliminates the influence of gene length and sequencing depth on the calculation of gene expression. Thus, the calculated gene expression can be directly used to compare gene expression between samples.

### Sequence and phylogenetic analysis

The open reading frame (ORF) of the putative OBP genes was predicted using ORF Finder (http://www.ncbi.nlm.nih.gov/gorf/gorf.html). The putative signal peptides of the amino acid sequences were predicted using the SignalIP 4.1 server (http://www.cbs.dtu.dk/services/SignalP/). The 38 OBP amino acid sequences were aligned using ClustalW [44] and edited using BioEdit Sequence Alignment Editor 7.1.3.0 [45].

A total of 192 OBP protein sequences (S3 Table) from 24 different Hemiptera insects were used for the phylogenetic analysis, including 38 *A*. *lucorum* OBPs identified in present study; seven OBPs from *N*. *lugens*, nine OBPs from *S*. *furcifera*, nine OBPs from *Laodelphax striatella*, one OBP from *Aphis craccivora*, two OBPs from *Aphis fabae*, nine OBPs from *Aphis glycines*, 11 OBPs from *Acyrthosiphon pisum*, eight OBPs from *A*. *gossypi*, one OBP from *Brevicoryne brassicae*, one OBP from *Drepanosiphum platanoidis*, six OBPs from *Metopolophium dirhodum*, five OBPs from *Megoura viciae*, five OBPs from *Myzus persicae*, four OBPs from *Nasonovia ribis-nigri*, five OBPs from *Pterocomma salicis*, three OBPs from *Rhopalosiphum padi*, seven OBPs from *Sitobion avenae*, one OBP from *Tuberolachnus salignus*, 14 OBPs from *A*. *lineolatus*, 32 OBPs from *L*. *lineolaris*, six OBPs from *Adelphocoris suturalis*, four OBPs from *Rhodnius prolixus*, and four OBPs from *Euschistus heros* [25–30, 46]. Species abbreviations: Aluc—*A*. *lucorum*, Alin—*A*. *lineolatu*s, Llin—*L*. *lineolaris*, Asut—*A*. *suturalis*, Rpro—*R*. *prolixus*, Eher—*E*. *heros*; Acra—*A*. *craccivora*, Afab—*A*. *fabae*, Agly—*A*. *glycines*, Agos—*A*. *gossypii*, Apis—*A*. *pisum*, Bbra—*B*. *brassicae*, *Dpla—D*. *platanoidis*, Mdir—*M*. *dirhodum*, Mvic—*M*. *viciae*, Mper—*M*. *persicae*, Nrib—*N*. *ribis-nigr*, Psal—*P*. *salicis*, Rpad—*R*. *padi*, Save—*S*. *avenae*, Tsal—*T*. *salignus*; Nlug—*N*. *lugens*, Sfur—*S*. *furcifera*, *Lstr—L*. *striatella*. A neighbor-joining tree was constructed using the MEGA 5.0 program [47] with default settings and bootstrap support based on 1000 iterations.

### qRT-PCR measurement

The relative transcript abundance of odorant binding protein genes in antennae, stylets, heads without antennae and stylets, thoraxes, abdomens, legs and wings from specimens of both genders were further quantified through qRT-PCR using the ABI Prism 7500 Fast Detection System (Applied Biosystems, Carlsbad, CA, USA). The cDNA from all tissues were synthesized using the Fast Quant RT kit (TIANGEN, Beijing, China). The reference gene GAPDH (GenBank accession No. JX987672) was used for normalization. The primers of the target and reference genes were designed using the Primer 3 program (http://frodo.wi.mit.edu/) and are listed in S4 Table. The specificity of each primer set was validated using a melt-curve analysis and the efficiency was calculated after analyzing the standard curves using a five-fold cDNA dilution series. The qRT-PCR reactions were conducted in a 20 μl mixture containing 10 μl of 2 × SuperReal PreMix Plus (TianGen, Beijing, China), 0.6 μl of each primer (10 μM), 0.4 μl of 50 × Rox Reference Dye, 1 μl of sample cDNA and 7.4 μl of sterilized H_2_O. The qRT-PCR cycling parameters included 95°C for 15 min, followed by 40 cycles of 95°C for 10 s and 62°C for 30 s, with melt curve stages at 95°C for 15 s, 60°C for 1 min, and 95°C for 15 s. Negative controls without either template were included in each experiment. Each reaction was performed with three biological replicates and each biological replicate was assessed three times. The comparative 2^-ΔΔCT^ method [48] was used to calculate the relative transcript levels in each tissue sample. Data were analyzed using SPSS Statistics 18.0 software (SPSS Inc., Chicago, IL, USA). ANOVA and Duncan's new multiple range test (*P* < 0.05) were used to compare the expression of each target gene among various tissues.

## Results

### Identification of *A*. *lucorum* OBPs

The tBLASTn results showed 33 unigenes encoding putative OBP genes in the *A*. *lucorum* antennal transcriptome (Table 1). Among the 33 *A*. *lucorum* OBPs (AlucOBPs), 10 OBPs (AlucOBP2-9, 11 and 12) were reported in our previous study [30]. 30 of the 33 AlucOBPs had intact ORFs with lengths ranging from 390 bp to 642 bp, all the 30 full-length AlucOBPs had signal peptide at their N-terminal (Table 1). Three AlucOBPs (AlucOBP2, 11 and 28) are partial with the N-terminal missing. The nucleotide sequences of all the 33 AlucOBPs were confirmed by cloning and sequencing. We also obtained five other AlucOBP genes by gene cloning method (AlucOBP1, 10 and 36–38) (Table 1).

**Table 1:** Odorant-binding proteins in *A*. *lucorum*.

Gene name	Accession Number	Length (AA)	Signal peptide	RKPM		Best BLASTx hit					
				Male	Female	Gene annotation	Species	Protein ID	Score (bits)	E-value	Identify
AlucOBP1	HQ631397	194	1–18	—	—	odorant-binding protein 12	*Apolygus lucorum*	AFJ54053.1	137	2.E-36	40%
AlucOBP2	HQ631398	159	Not detected	61	43	odorant-binding protein 18a	*Lygus lineolaris*	AHF71046.1	308	1.E-104	93%
AlucOBP3	HQ631399	203	1–18	23	20	odorant-binding protein 25	*Lygus lineolaris*	AHF71056.1	195	8.E-59	57%
AlucOBP4	HQ631400	181	1–19	10	10	odorant-binding protein 28	*Lygus lineolaris*	AHF71059.1	309	3.E-104	93%
AlucOBP5	HQ631401	174	1–19	1	6	odorant-binding protein 30	*Lygus lineolaris*	AHF71061.1	279	1.E-92	86%
AlucOBP6	HQ631402	189	1–19	2	2	odorant-binding protein 24	*Lygus lineolaris*	AHF71055.1	209	1.E-65	85%
AlucOBP7	JQ675724	145	1–18	22397	16321	odorant-binding protein 8	*Lygus lineolaris*	AHF71035.1	234	6.E-76	83%
AlucOBP8	JQ675725	149	1–24	3795	3795	odorant-binding protein 2	*Lygus lineolaris*	AHF71029.1	284	2.E-95	93%
AlucOBP9	JQ675726	153	1–19	0.6	1	odorant-binding protein 19	*Lygus lineolaris*	AHF71049.1	211	1.E-66	65%
AlucOBP10	JQ675727	156	Not detected	—	—	odorant-binding protein 5	*Apolygus lucorum*	AEP95759.1	204	6.E-64	84%
AlucOBP11	JQ675728	199	Not detected	125	58	odorant-binding protein 6	*Apolygus lucorum*	AEA07664.1	72	3.E-12	29%
AlucOBP12	JQ675729	194	1–20	258	248	odorant-binding protein 1	*Apolygus lucorum*	AEA07705.1	137	2.E-36	43%
AlucOBP13	KT281921	145	1–22	1925	996	odorant-binding protein 12	*Lygus lineolaris*	AHF71039.1	246	6.E-80	79%
AlucOBP14	KT281922	142	1–21	69	74	odorant-binding protein 3	*Lygus lineolaris*	AHF71030.1	257	6.E-85	94%
AlucOBP15	KT281923	143	1–27	115	113	odorant-binding protein 15	*Lygus lineolaris*	AHF71042.1	200	1.E-62	74%
AlucOBP16	KT281924	149	1–19	8	6	odorant-binding protein 16a	*Lygus lineolaris*	AHF71043.1	231	2.E-74	73%
AlucOBP17	KT281925	132	1–16	40931	23520	antennal protein LAP	*Lygus lineolaris*	AAC43033.1	246	8.E-81	90%
AlucOBP18	KT281926	144	1–19	29	38	odorant-binding protein 11	*Lygus lineolaris*	AHF71038.1	274	1.E-91	93%
AlucOBP19	KT281927	152	1–20	1207	1222	odorant-binding protein 8	*Lygus lineolaris*	AHF71035.1	147	9.E-42	50%
AlucOBP20	KT281928	194	1–18	0.4	0.6	odorant-binding protein 20	*Lygus lineolaris*	AHF71050.1	197	9.E-61	74%
AlucOBP21	KT281929	148	1–20	3369	2888	odorant-binding protein 6	*Lygus lineolaris*	AHF71033.1	240	3.E-78	97%
AlucOBP22	KT281930	138	1–20	25774	26318	odorant-binding protein 4	*Lygus lineolaris*	AHF71031.1	273	2.E-91	95%
AlucOBP23	KT281931	213	1–18	501	637	odorant-binding protein 23	*Lygus lineolaris*	AHF71053.1	258	3.E-83	74%
AlucOBP24	KT281932	155	1–21	5048	5063	odorant-binding protein 5	*Lygus lineolaris*	AHF71032.1	293	1.E-98	88%
AlucOBP25	KT281933	204	1–19	2	6	odorant-binding protein 25	*Lygus lineolaris*	AHF71056.1	287	9.E-95	76%
AlucOBP26	KT281934	145	1–18	1734	1352	odorant-binding protein 10	*Lygus lineolaris*	AHF71037.1	196	4.E-61	70%
AlucOBP27	KT281935	154	1–26	1817	1879	odorant-binding protein 6	*Apolygus lucorum*	AEA07664.1	179	3.E-54	60%
AlucOBP28	KT281936	155	Not detected	55	58	odorant-binding protein 10	*Adelphocoris lineolatus*	ACZ58081.1	237	1.E-76	72%
AlucOBP29	KT281937	202	1–17	253	212	odorant-binding protein 29	*Lygus lineolaris*	AHF71060.1	343	6.E-117	95%
AlucOBP30	KT281938	166	1–19	2	1	odorant-binding protein 30	*Lygus lineolaris*	AHF71061.1	156	2.E-44	54%
AlucOBP31	KT281939	205	1–19	2	2	odorant-binding protein 25	*Lygus lineolaris*	AHF71056.1	204	3.E-62	67%
AlucOBP32	KT281940	182	1–18	0.7	0.4	odorant-binding protein 32	*Lyguslineolaris*	AHF71063.1	136	5.E-37	45%
AlucOBP33	KT281941	155	1–23	6	7	odorant-binding protein 3	*Euschistus heros*	AIU64820.1	68.9	1.E-11	32%
AlucOBP34	KT281942	158	1–24	23	24	odorant-binding protein 9	*Adelphocoris lineolatus*	ACZ58080.1	201	1.E-62	69%
AlucOBP35	KT281943	129	1–17	9	6	odorant-binding protein 6	*Spodoptera exigua*	AFM77984.1	52.4	4.E-06	27%
AlucOBP36	KT281944	150	1–19	—	—	odorant-binding protein 11	*Adelphocoris suturalis*	AHJ81243.1	298	6.E-101	97%
AlucOBP37	KT281945	152	1–20	—	—	odorant-binding protein 8	*Adelphocoris lineolatus*	ACZ58079.1	309	5.E-105	98%
AlucOBP38	KT281946	153	1–27	—	—	odorant-binding protein 6	*Adelphocoris suturalis*	AHJ81241.1	304	3.E-103	99%

“—” Genes were obtained by gene cloning and the RPKM values can not be calcuated.

**Fig 1:**
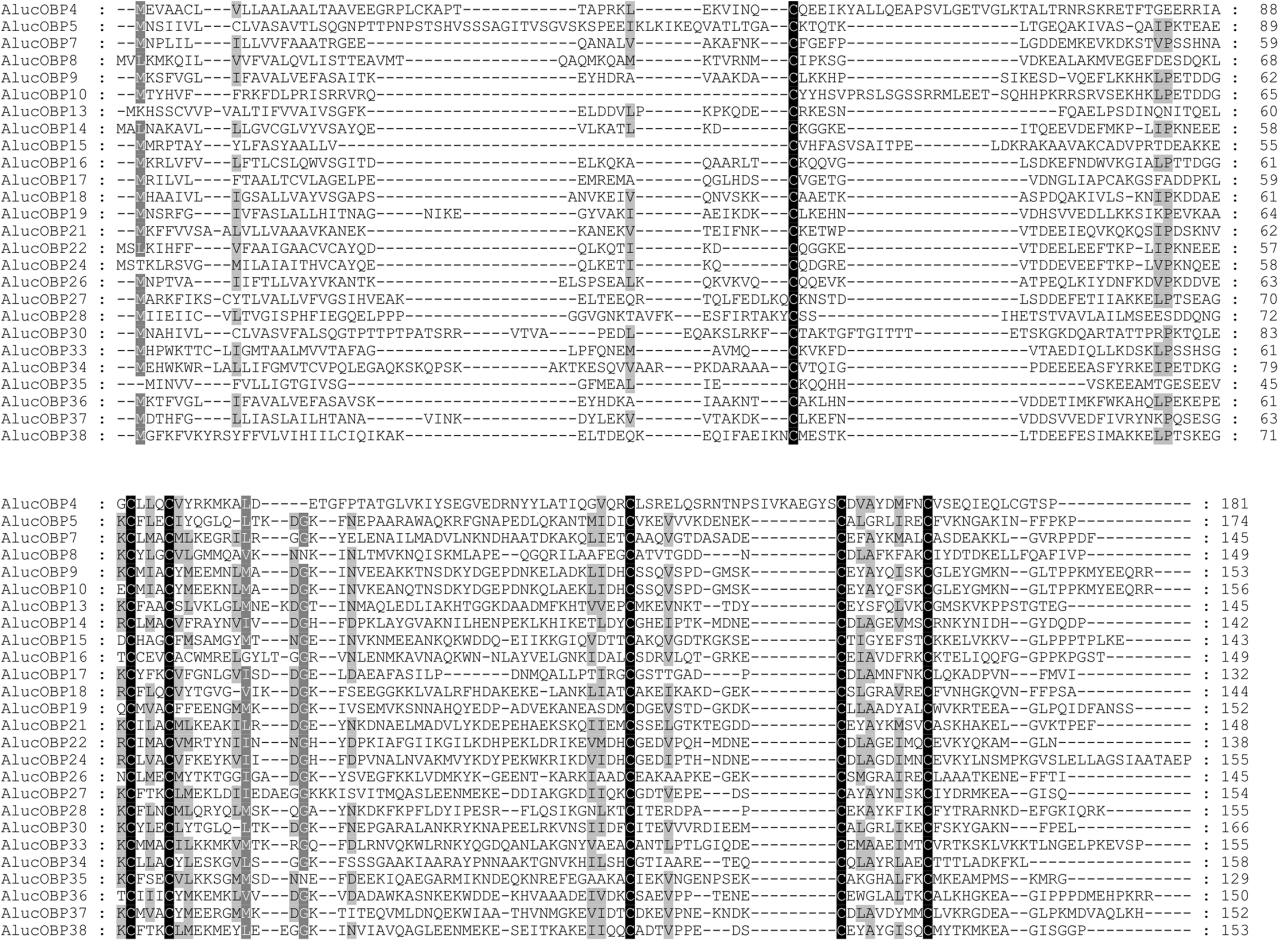
Alignment of *A*. *lucorum* ‘classic’ odorant-binding proteins (OBPs). Sequences were aligned using the program ClustalW and further edited using BioEdit Sequence Alignment Editor 7.1.3.0. The conserved Cys residues (C1-C6) in the ‘classic’ OBP motif are indicated. Shading represents conservation of sequence identity.

All 38 complete AlucOBP sequences containing a significant portion of the characteristic OBP Cys signature were categorized into two types, ‘classic’ OBP and ‘Plus-C’ OBP. Based on the Hemipteran ‘classic’ OBP Cys motif (C1-X_22-32_-C2-X_3_-C3-X_36-46_-C4-X_8-14_-C5-X_8_-C6) [49–51], we classified 26 AlucOBP (AlucOBP4, 5, 7–10, 13–19, 21, 22, 24, 26–28, 30 and 33–38) sequences as ‘classic’ OBPs (Fig 1). The remaining 12 AlucOBPs (AlucOBP1-3, 6, 11, 12, 20, 23, 25, 29, 31 and 32) belong to the ‘Plus-C’ OBP family, and fit to the Cys spacing pattern.

**Fig 2:**
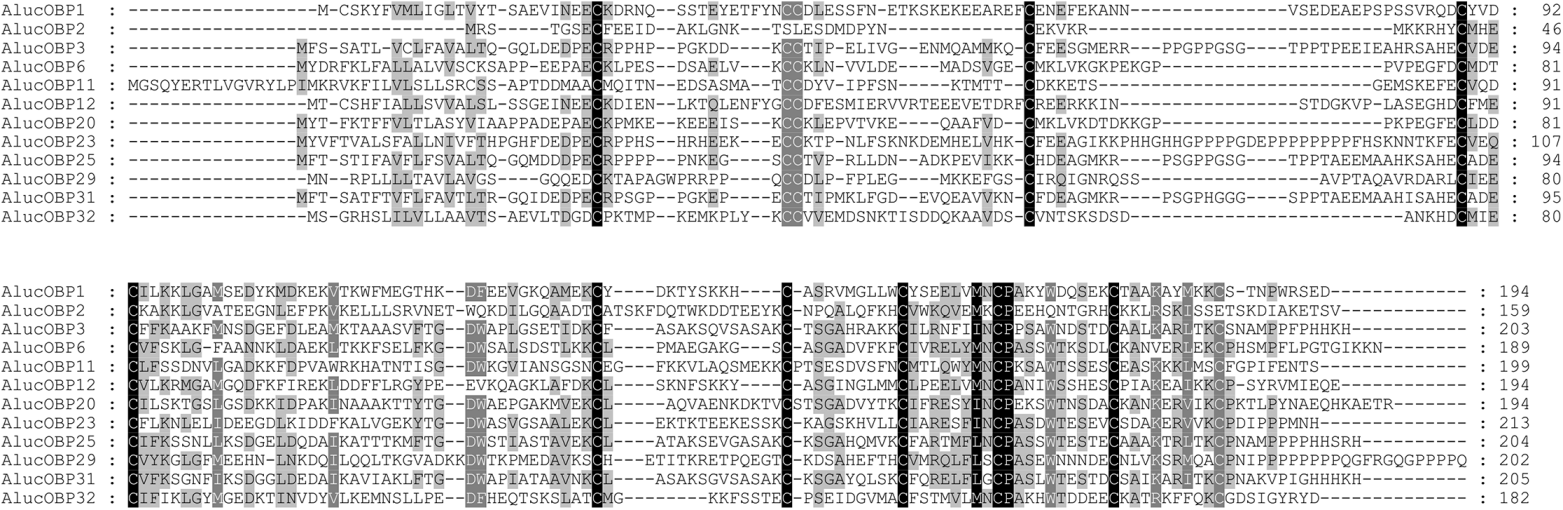
Alignment of *A*. *lucorum* ‘Plus-C’ odorant-binding proteins (OBPs). Sequences were aligned using the program ClustalW and further edited using the BioEdit Sequence Alignment Editor 7.1.3.0.The conserved residues in the ‘Plus-C’ OBP motif are indicated. Shading represents conservation of sequence identity.

C1-X_8-41_-C2-X_3_-C3-X_39-47_-C4-X_17-29_-C4a-X_9_-C5-X_8_-C6-P-X_9-11_-C6a [51] (Fig 2).

The 38 OBPs within *A*. *lucorum* shared relative low amino acid identities (3.8%–66.8%) with each other (S5 Table), but each of the AlucOBPs had a very high amino acid identity with the homologous OBPs in two closed species *L*. *lineolaris* and *A*. *lineolatus*, for example, AlucOBP21 showed as high as 97% identity with LlinOBP6 (Table 1).

### Phylogenetic analysis of AlucOBP sequences

**Fig 3:**
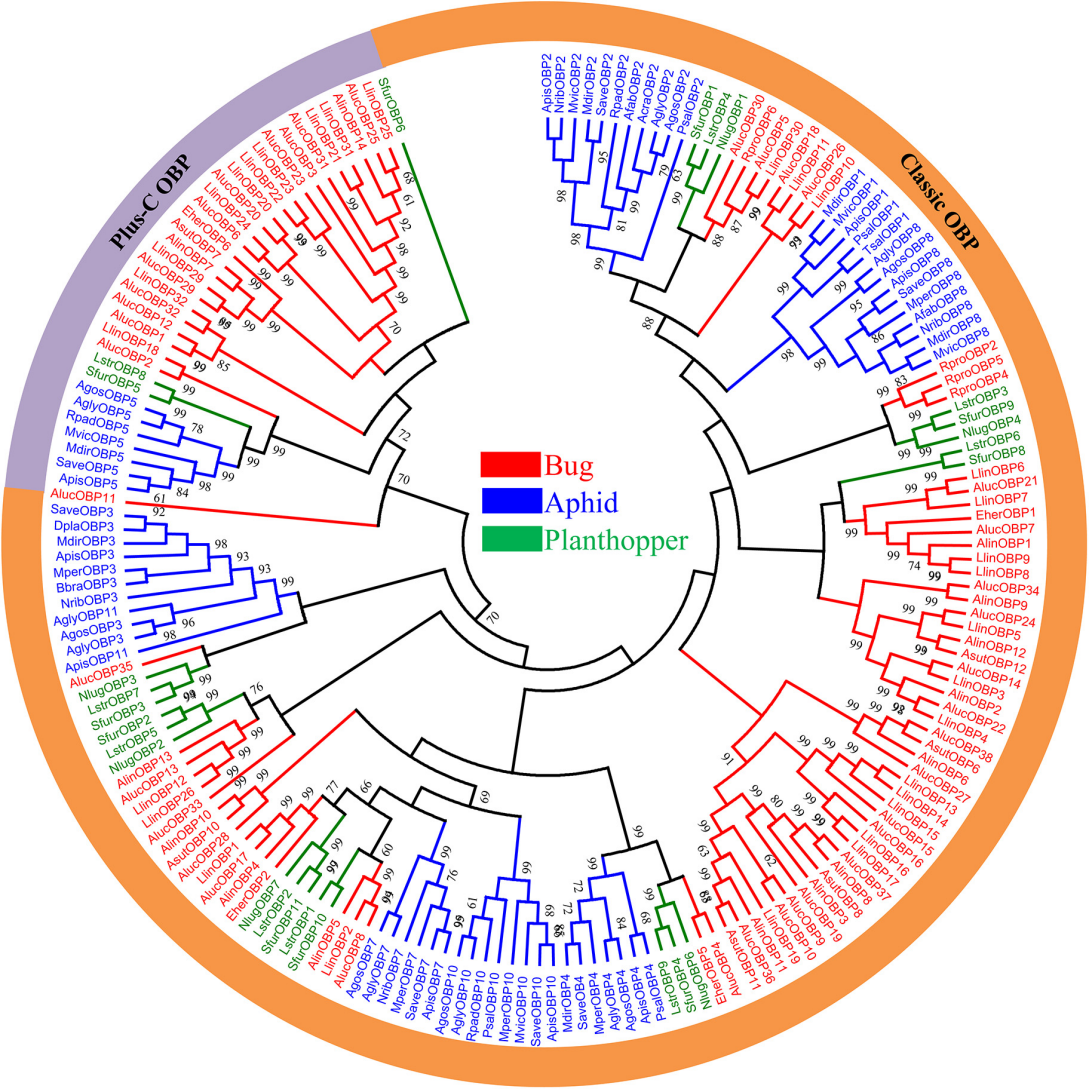
Phylogenetic relationship of 192 odorant-binding proteins (OBPs) from 24 Hemipteran species. Sequences were aligned using MAFFT and the phylogenetic tree constructed using MEGA5 with bootstrap support based on 1000 iterations (only bootstrap values > 60% are shown). Bug sequences are shown in red, AlucOBP sequences are shown in bold italic; Aphid sequences are shown in blue; Planthopper sequences are shown in green.

A neighbor joining tree of 192 OBPs was constructed from four plant bugs and 20 other Hemipteran species (Fig 3). The Hemipteran OBP protein family generates an expansive tree, with ‘classic’ OBP and ‘Plus-C’ OBP sequences segregating into unique clades. In the phylogenetic tree, OBPs belonging to the same family (bug, aphid and planthopper OBPs) cluster together locally in the phylogenetic tree, but the same family OBPs segregate into different central clusters, distributing equally throughout the entire phylogenetic tree. These results suggest that Hemipteran OBP proteins undergo extensive gene duplication and divergence. OBPs in same species are divergent from each other, further demonstrating the diversity of OBP gene family, consistent with previous reports regarding the lack of overall OBP sequence conservation [13, 25, 26, 46, 50, 52, 53].

There are many orthologous sequences in the *A*. *lucorum* OBPs which are largely limited to *L*. *lineolaris* OBPs with a high bootstrap value, for example AlucOBP34 and AlinOBP9 located in the same branch with the bootstrap value as high as 99. This finding suggests that these sequences originate from the same ancestors, vertically descend, and are conserved for common functions in plant bug species. However, some AlucOBPs have no orthologous sequences. This finding is somewhat different from aphids which have most orthologous sequences in different species. AlucOBP genes also have paralogs, such as AlucOBP1 with AlucOBP12 and AlucOBP9 with AlucOBP10, which potentially were horizontally duplicated from the same ancestor through natural selection to obtain an additional function.

### Expression profiles of the AlucOBP genes

**Fig 4:**
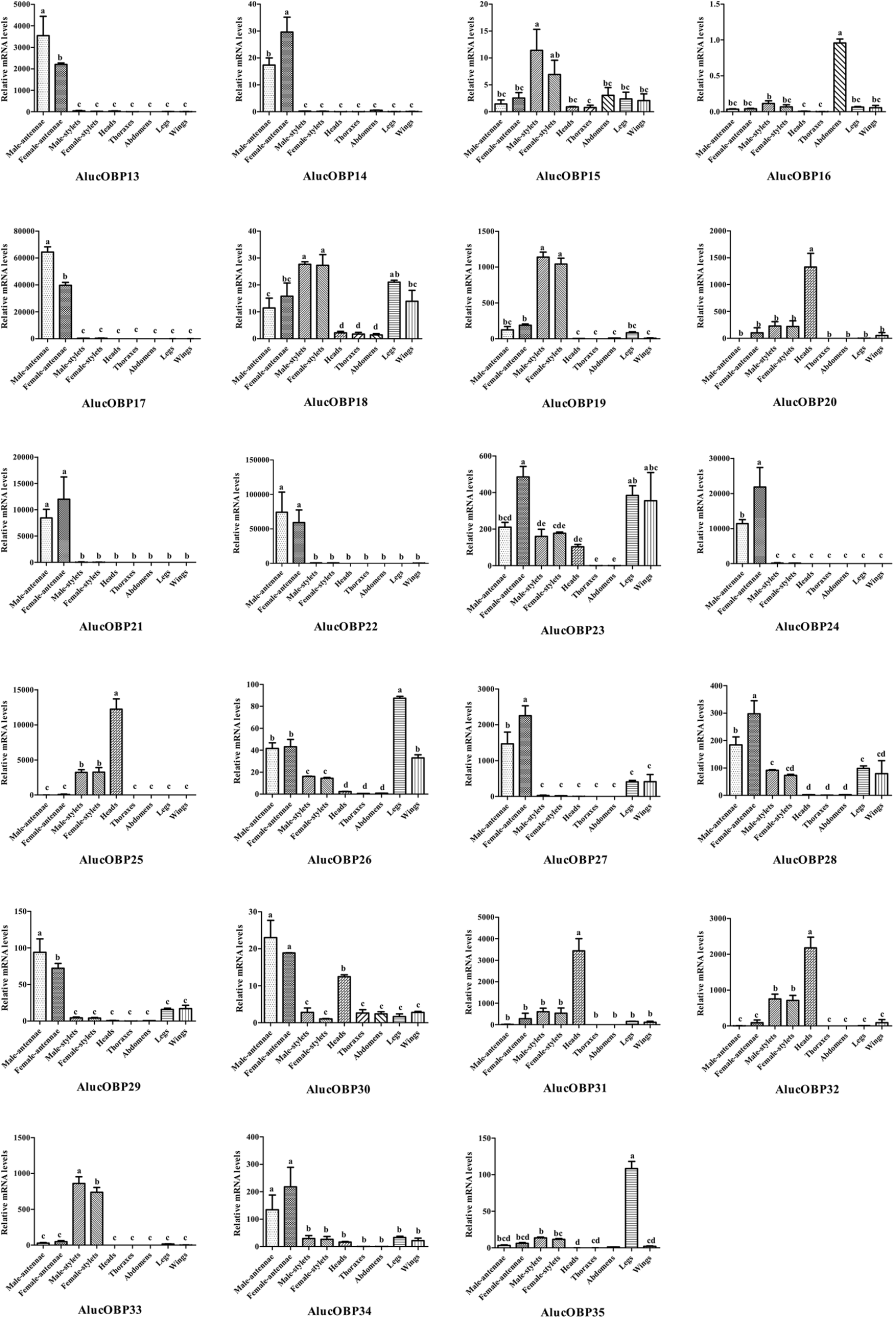
Transcript abundances of *A*. *lucorum* OBP genes. Transcription levels of OBP genes were normalized by GAPDH, and normalized transcript levels to that of male abdomen. The error bar represents standard error and the different small letters above each bar indicate significant differences in transcript abundances (*p* < 0.05).

To further characterize the AlucOBPs, we used qRT-PCR to measure the expression profiles of the 23 AlucOBPs which were reported for the first time in different tissues such as antennae, stylets, heads (without antennae and stylets), thoraxes, abdomens, legs and wings (Fig 4). Insect OBPs are typically the most highly expressed genes in peripheral olfactory tissues including the antennae and other parts of the body [13].

The expressions of 11 OBP genes (AlucOBP13, 14, 17, 21, 22, 24, 27–30 and 34) were significantly higher in the antennae of both sexes than in other tissues. Furthermore, among the 11 antennae-enriched AlucOBPs, three AlucOBPs (AlucOBP13, 17 and 29) are significantly male antennae-biased, and six AlucOBPs (AlucOBP14, 21, 24, 27, 28 and 34) are significantly female antennae-biased. The RPKM value analysis demonstrated a certain degree of sex-biased OBP expression in the antennae, consistent with the qRT-PCR results [43]. The OBPs were defined as highly expressed genes with RPKM values greater than 1000. RPKM measurements provide additional supporting evidence that some AlucOBPs (AlucOBP7, 8, 13, 17, 19, 21, 22, 24, 26 and 27) are highly expressed in the antennae based on RPKM values greater than 1000 (Table 1).

AlucOBP15, AlucOBP19 and AlucOBP33 showed significantly higher expression in stylets of one or both genders than in any other tissues. Both AlucOBP26 and AlucOBP35 had significantly higher expression in the legs of both genders than in any other tissues. AlucOBP20, AlucOBP25, AlucOBP31 and AlucOBP32 had significantly higher expression in the head of both sexes than in any other tissues. Interestingly, all the head-enriched OBP genes were more or less expressed in the stylets. AlucOBP16 showed relatively high expression in the abdomens. AlucOBP18 and AlucOBP23 were ubiquitously expressed in the main tissues, such as the antennae, stylets, heads, legs and wings.

Notably, AlucOBP17, AlinOBP4 and LlinOBP1, which are orthologous genes that cluster together in the clade described above, have similar expression patterns as these genes were all highly expressed in antennae [25, 26]. Not accidently, the most homologous genes (among AlucOBPs, AlinOBPs and LlinOBPs) had similar or even almost identical expression profiles in different tissues. Compared with the resemblance to the expression profile in orthologous genes, paralogous genes might have similar or different expression profiles, resulting in functional differences (AlucOBP1 with AlucOBP12) or conservation (AlucOBP9 with AlucOBP10) [25, 26].

## Discussion

In the present study, we first used a transcriptomics approach to identify 38 OBP-like transcripts (including five obtained through gene cloning), 23 of which were reported for the first time. The number of OBPs in *A*. *lucorum* was significantly higher as compared to those previously reported for other Hemipteran species: 15 genes in *A*. *pisum* [46], nine genes in *A*. *gossypii* [27], 10 genes in *N*. *lugens* [28] and 11 genes in *S*. *furcifera* [29]. Multiple factors are likely responsible for the differences in the OBP numbers. Studies have demonstrated a wide range of host plants and obvious seasonal alterations in host ranges, which might lead to an increased number of OBP genes to achieve more abundant and complex functions [4, 54], either through the potential long-distance flight capability and seasonal migration [55, 56], or as a result of a complicated and heterogeneous host environment, rather than the highly specialized ecology and parasitic lifestyle observed in *A*. *pisum* [46]. Moreover, the number of OBPs in *A*. *lucorum* is equal to 33 genes *in L*. *lineolaris*, as determined through the construction of a whole-body cDNA library comprising tissues from both sexes across all the developmental stages [25], but is more than 14 genes in *A*. *lineolatus*. Broadly overlapping host ranges and the same habitat contribute to the similarity observed in *A*. *lucorum* and *L*. *lineolaris*. In addition, due to limited ESTs, some OBPs have not yet been identified in *A*. *lineolatus* [26].

Insect OBPs are a super-gene family, with a high degree of intraspecific and interspecific sequence differentiation, as the AlucOBP repertoire shares relatively low amino acid identities among these genes. However, the high degree of sequence conservation between AlucOBPs and those in other plant bugs is consistent with the interspecies sequence similarity which were observed in aphids [27, 46]. Furthermore, as AlucOBPs homologous genes are largely limited to *L*. *lineolaris* OBPs, the sequences of several AlucOBPs proteins show a high degree of similarity with *L*. *lineolaris* OBPs. In addition, the highest degree of identity in the best BLASTx hit was observed with *L*. *lineolaris* OBP protein sequences. In previous studies (unpublished data), we observed that *Lygus pratenszs* (Linnaeus) is widely distributed in Xinjiang cotton fields, showing OBP genes identical to those of *L*. *lineolaris*, suggesting that these insects belong to the same species despite geographic isolation. Considering the similar life style and host relationship, mirid bugs have a close relationship not only in the olfactory gene OBPs, but also in perception of semiochemicals [57, 58].

The phylogenetic analysis revealed a divergent repertoire, and a large percentage of AlucOBP genes with orthologs. The distribution of AlucOBP orthologs in other mirid bug species, suggests that these genes originated from the same ancestors to perform similar functions or acquire different functions through undergoing subfunctionalization. The OBPs from four different mirid bugs clustered into scattered family branches as orthologous groups (for example AlucOBP24, LlinOBP5, AlinOBP12 and AsutOBP12), which has also been observed in Aphid OBPs, potentially demonstrated that the orthologous OBP genes in mirid bugs diverged before speciation, although some potential OBPs in mirid bugs (as AlinOBPs, AsutOBPs) have not been identified [27, 46]. Interestingly, the orthologous OBP groups in mirid bugs are less conserved than aphid OBPs with low identities in different species. Some paralogs in AlucOBPs clustered together in the same subgroup, suggesting that these OBPs also diverged from the same ancestors. The phylogenic tree also shows higher divergence times for paralogs compared with orthologs. The 38 AlucOBPs display a clear divergence through natural selection, suggesting that these genes have undergone subfunctionalization or neofunctionalization.

The potential biological function of OBPs can be derived from the expression profile using different methods, such as microarray hybridization and qRT-PCR [25, 26]. In Hemipteran insect OBPs, a high degree of expression was observed in the antennae. A total of 21 *L*. *lineolaris* OBPs, 12 *A*. *lineolatus* OBPs, five *A*. *gossypii* OBPs, more than six *N*. *lugens* OBPs and three *S*. *furcifera* OBPs were highly expressed in the antennae [25–29]. Among the AlucOBPs, 16 OBP genes were significantly more highly expressed in the antennae of female and/or male adults than in other tissues (5 previously identified AlucOBPs expression patterns referred to Ji and Hua [20, 35]). The high antennal expression of AlucOBPs is clearly correlated with a role in olfaction. Some of the AlucOBP genes showed high RPKM values, reflecting considerable abundance in antennal cDNA libraries, which is consistent with high expression in *A*. *lucorum* antennae, as confirmed through qRT-PCR. The expression profile also demonstrated a certain degree of sex-biased OBP expression in the chemosensory antennae tissue. The male-dominant expression of OBPs might function in the detection and discrimination of sex pheromones, while the female-dominant expressions of OBPs might have the preferential function of locating the host plant and detecting egg-laying substrates. Surprisingly, less sex bias was observed for almost all AlucOBPs in non-antennal tissues.

The antennal-specific protein (LAP) of the tarnished plant bug *L*. *lineolaris* was the first OBP identified in Hemipteran [59], and this protein was only detected in male and female antennae through immunoblotting [60] and in situ hybridization [54]. Subsequent studies have reported increasing evidence that OBPs not only show antennae-specific expression, but are also expressed in other tissues, such as heads, stylets, legs and wings [25, 26, 29]. In the present study, three OBP genes showed significantly higher expression in the stylets of one or both sexes. Four OBPs showed significantly higher expression in the heads of both genders, meanwhile, all these head-enriched OBPs were more or less expressed in stylets. This finding might reflect the presence of gustatory organs on the head, such as salivary glands, correlating with stylets, which participate together in taste perception. It is also possible that these AlucOBPs could be expressed in the brain, correlated with the delivery of neurotransmitters. However, further studies are required. In general, OBP expression in the gustatory organs on the head, such as the proboscis and labellum, could be associated with the gustatory response. In *D*. *melanogaster*, PBPRP2 was abundantly expressed in adult gustatory organs on the labellum and in the internal taste organs on the proboscis, potentially acting as a carrier for bitter tastants [19]. OBP49a was highly enriched in gustatory sensilla on the main taste organ, the labellum, with an unexpected role in taste and as a molecular player involved in the integration of opposing attractive and aversive gustatory inputs [21]. Similar to the gustatory aspects of the proboscis and potential gustatory organs on the head, OBP transcript expression in legs and wings may be indicative of gustatory function. In our research, some AlucOBPs were highly expressed in the legs or wings, suggesting that these genes might participate in taste functions. *D*. *melanogaster* OBPs were also expressed in gustatory organs on the wings and legs [18, 19]. In previous studies of AlucOBPs, three putative OBP genes (AlucOBP36, 37 and 38) were primarily expressed in the antennae, proboscis and legs. The high affinities with cotton secondary metabolites rather than plant volatiles, suggests roles of these OBPs in gustation rather than in olfaction [20]. Remarkably, AlucOBP38 could strongly bind with toxic cotton secondary metabolites, such as gossypol.

Enrichment in the abdomen has been associated with other physiological functions. *A*. *aegypti* OBP22 was expressed not only in sensory organs, such as the antennae and proboscis, but also in the male reproductive apparatus and transferred to the spermathecs, at the tip of the female abdomen, suggesting an additional function for this protein as a pheromone transporter. *A*. *aegypti* OBP22 was also expressed in the thorax, in the insect respiratory system [61]. AlucOBP5 was highly expressed in the female abdomen and male legs, suggesting that the female *A*. *lucorum* might use AlucOBP5 for storing and releasing chemical compounds from special glands in the abdomen, while in the male *A*. *lucorum*, AlucOBP5 might be involved in chemoreception in the sensilla of the legs [62].

Previous studies revealed that in sibling species conserved OBP genes, similar expression locations and physiological functions were observed for corresponding proteins. For example, aphid OBP3 and OBP7 mediate behavioral responses to the alarm pheromone *E*-*β*-farnesene [63]. In *H*. *armigera* and *H*. *assulta*, the conserved OBP10 also demonstrated expression in similar organs, such as antennae and reproductive organs, as a function of a carrier for the oviposition deterrent 1-dodecene [64]. In this work, most AlucOBP genes showed expression patterns similar to orthologous genes in other mirid bugs. The similar expression patterns of orthologs strongly indicate that these genes have the same or similar function. AlinOBP13 with AlucOBP13 and AlinOBP10 with AlucOBP22 have two pairs of homologous genes clustered in a branch in the phylogenetic tree with similar expression patterns, and these genes were both antennae-specific and highly expressed in the antennae. The results of a fluorescence displacement binding assay and electroantennogram recordings, showed that recombinant AlinOBP13 exhibited a more specific binding preference to terpenoids than other semiochemicals, and all of the tested terpenoids could elicit EAG responses at varying degrees in both male and female antennae [37]. The host plants of *A*. *lineolatus*, such as cotton and alfalfa, produce most of these terpenoids naturally or upon herbivore attack [65]. In addition, some terpenoids typically function as cues for some herbivores and their natural enemies to locate hosts or prey [66]. Thus, it is likely that AlucOBP13 might exhibit similar binding specificities. Furthermore, AlinOBP10 exhibited higher binding affinities not only to terpenoids but also to plant green leaf volatiles which are important signal volatiles and play key roles in insect-plant interaction [67], and 3-hexanone, an important volatile in cotton. Electroantennogram recordings and Y-tube olfactometer trials indicated that the plant green leaf volatiles (*E*)-2-hexenal and 1-hexanol and the cotton volatile 3-hexanone all elicited strong electrophysiological responses in the antennae of both sexes, and only 3-hexanone was significantly attractive, while (*E*)-2-hexenal and 1-hexanol were more repellent to *A*. *lineolatus* in behavioral trials [9].

*A*. *lucorum* adults use different types of host-related volatile and sex pheromone cues for host locations, host alterations and mating. Mirid sex pheromones could be used as potential biological agents to control plant bugs through mating disruption or mass trapping strategies [68]. Increasing evidence has shown that female mirids use sex pheromones to attract males [69]. The high expression of OBPs in male antennae might be important in the perception of sex pheromones, while the high expression of OBPs in female antennae might be implicated in the recognition of particular chemicals (such as aromatic compounds) released from flowering host plants to forage suitable hosts and oviposition sites and mediate seasonal alterations in a range of host, providing a starting point for using OBPs as targets to regulate the insect behavior of mating, feeding and oviposition and further to develop novel crop protection strategies.

Using a transcriptomics-based approach, we expanded the current understanding of the *A*. *lucorum* olfactory system, identifying 23 additional OBPs. Based on the results of the transcript expression profile, future studies will focus on the binding function of candidate OBPs with the identified sex pheromone components, such as 4-oxo-(*E*)-2-hexenal and trans-2-hexenyl butyrate, which have been used to trap *A*. *lucorum* adults or interfere with mating in the field and some significant aromatic plant volatiles detected from prefered host plants of *A*. *lucorum*. In addition, stylet- and head-based gestation and the role of OBPs might reflect gustatory-driven behaviors and these hypotheses require further study.

## Supporting Information

S1 Table Primers used for gene cloning. (DOCX)

S2 Table Primers used for gene cloning and sequencing. (DOCX)

S3 Table Accession numbers for amino acid sequences of OBPs in phylogenetic tree. (DOCX)

S4 Table Primers used for qRT-PCR. (DOCX)

S5 Table Comparison of *A*. *lucorum* OBP proteins sequences. (XLSX)
