# Supplementary material for: Molecular Characterization and Expression Profiling of Odorant-Binding Proteins in Apolygus lucorum
Source: PLoS One. 2015 Oct 14;10(10):e0140562. doi: 10.1371/journal.pone.0140562 (PMC4605488; doi:10.1371/journal.pone.0140562)
Supplement: S1 Table — (DOCX) [file pone.0140562.s001.docx]

**Supplementary materials**

**S1 Table. Primers used for gene cloning**

| **Genes** | **Forward (5'-3')** | **Reverse (5'-3')** |
| --- | --- | --- |
| AlucOBP1 | ATGTGTTCAAAATACTTTGTTATGC | TCAATCTTCACTCCTCCATGGG |
| AlucOBP10 | ATGACTTACCATGTGTTCTTTCGAA | TCAGCGTCTTTGTTCTTCGTACA |
| AlucOBP36 | ATGAAAACCTTCGTAGGACTCATCT | TTATCGCCTTTTTGGGTGTTC |
| AlucOBP37 | ATGGACACACATTTCGGGTTG | TCAGTGCTTGAGCTGCGCA |
| AlucOBP38 | ATGGGCTTCAAGTTCGTCAAATA | TCAGGGCCCCCCCGAAATT |
