# Supplementary material for: Molecular Characterization and Expression Profiling of Odorant-Binding Proteins in Apolygus lucorum
Source: PLoS One. 2015 Oct 14;10(10):e0140562. doi: 10.1371/journal.pone.0140562 (PMC4605488; doi:10.1371/journal.pone.0140562)
Supplement: S2 Table — (DOCX) [file pone.0140562.s002.docx]

**Supplementary materials**

**S2 Table. Primers used for gene cloning and sequencing**

| **Genes** | **Forward (5'-3')** | **Reverse (5'-3')** |
| --- | --- | --- |
| AlucOBP2 | AGCACGGGAAAAATGAATCTATG | ACAGGAAGAGCTTCTCAAACGC |
| AlucOBP3 | TGGATTCATCAATGTTCTCCTCTG | CGGGTGAAACTTGAATCTAATGTT |
| AlucOBP4 | ATGGAGGTTGCAGCTTGCC | CTAGGGAGACGTCCCGCAA |
| AlucOBP5 | TAACTCGTGCAACATGAACTCCA | CCTGCTATTCACGGCTTGG |
| AlucOBP6 | GAAGGACTTCAGACGGCCATA | TCATTTTCAGTTCTTTTTGATTCCT |
| AlucOBP7 | ATCTACAAGCGACATCATGAACC | CGGTGTGTTTTCAGAAATCTGG |
| AlucOBP8 | AATACTTGGTGTCACCAGGGGA | TCTGACTTAGTTTTTCATGGAACAAT |
| AlucOBP9 | ATGAAGTCTTTCGTAGGTTTGATCT | CCGTTCTGGTTCAGCGTCTT |
| AlucOBP11 | CTCGACATGGGATCTCAGTATGA | GTTCATGATGTGTTCTCGAATATTG |
| AlucOBP12 | TAATCGTCGTTCCCAACATGA | TGCAGCAGTTCATAGCTATTCTTG |
| AlucOBP13 | TATTGATCGTAACAATTTTTGCG | GTTGCTAAAAATGGTTATCCTTCTG |
| AlucOBP14 | TACAGAATTTCAGGAAATGGCTCT | GTGTCTAGGGGTCTTGATCGTATC |
| AlucOBP15 | CTCACTACTTCGACGATCATGATG | AAGTCACTCCTTACTCCTTTAAAGG |
| AlucOBP16 | ACCTCTTCGTCTCATCATGAAGAG | ACCCTCATGTTGAGCCTGGT |
| AlucOBP17 | CTCTCTACCCAGCCGAAATGAG | TTATGTAATTAGATCACCATGAAGTTCA |
| AlucOBP18 | CCGTCTCCCTCAACCAATCAT | AGATCGGGGTTTTACGCAGAT |
| AlucOBP19 | AAAGTGAACGTCATGAACTCTCGT | ATCAGGAGGCGTTAGCAAAGTC |
| AlucOBP20 | ATGTACACATTCAAGACCTTTTTCGT | AGTACGTAGTTCACCGAGTTTCG |
| AlucOBP21 | ATCATCCTCATCATGAAGTTCTTC | TTTAGAATTCGGGGGTCTTGAC |
| AlucOBP22 | ATACCAACGAAGTCACACTATGTCTCT | GGTGAAGTGTGTCTCAGTTAAGACC |
| AlucOBP23 | GTGATCGTTCAAACATGTACGTCTT | GGTTCATCGGTGGGGGAAT |
| AlucOBP24 | TTCCTCCGTTCCAGAAATGTC | GTTTTGATCAGGGCTCGGC |
| AlucOBP25 | CGACATGTTTACTTCCACAATTTTC | ACTCGAACTAATGCCTGCTGTGA |
| AlucOBP26 | ACAGCAACAAACATGAATCCCAC | CAGTATTTTTTCTATATGGTGAAGAATTC |
| AlucOBP27 | GGCAGGATGGCCCGCA | GGAAAGTATTATTGAGAAATGCCTG |
| AlucOBP28 | AACGACATCTTGAAATCATGATCAT | TTTGCAGACATTATTTCCTCTGAAT |
| AlucOBP29 | TCACACCGAATCATGAATCGT | GTTGTTGATTGGTAACTGATCACTGA |
| AlucOBP30 | TACTCGAGCACCATGAACGC | TGCTATTCACAGCTCGGGAAA |
| AlucOBP31 | CTCCCACAACATGTTCACCTCT | TGAAGTGAAGTTCGAATCAATGTTT |
| AlucOBP32 | CAGAGTTCGTCATGAGTGGTCGT | TGTTCATTTTCAATCATAACGGTAAC |
| AlucOBP33 | ATGCACCCCTGGAAAACCAC | TACAGCAAGACTAAGGACTCACTTCC |
| AlucOBP34 | TACTCTCAGCAAGTGGTGAAAATG | AGCACCTGAATACACTAAAGTTTGAAT |
| AlucOBP35 | CATGATCAACGTCGTTTTCGTT | AGAAAACATTAACCCCGCATT |
