# Supplementary material for: Molecular Characterization and Expression Profiling of Odorant-Binding Proteins in Apolygus lucorum
Source: PLoS One. 2015 Oct 14;10(10):e0140562. doi: 10.1371/journal.pone.0140562 (PMC4605488; doi:10.1371/journal.pone.0140562)
Supplement: S3 Table — (DOCX) [file pone.0140562.s003.docx]

**Supplementary materials**

**S3 Table. Accession numbers for amino acid sequences of OBPs in phylogenetic tree**

| **Species** | **Gene name** | **Acc. number** | **Species** | **Gene name** | **Acc. number** |
| --- | --- | --- | --- | --- | --- |
| ***Apolygus lucorum*** | AlucOBP1 | AEA07705 |  | EherOBP5 | AIU64822 |
|  | AlucOBP2 | AEA07706 |  | EherOBP6 | AIU64823 |
|  | AlucOBP3 | AEA07661 | ***Tuberolachnus salignus*** | TsalOBP1 | CAR85659 |
|  | AlucOBP4 | AEA07662 | ***Sogatella furcifera*** | SfurOBP1 | KF732013 |
|  | AlucOBP5 | AEA07663 |  | SfurOBP2 | KF660218 |
|  | AlucOBP6 | AEA07664 |  | SfurOBP3 | KF732014 |
|  | AlucOBP7 | AFJ54048 |  | SfurOBP4 | KF732015 |
|  | AlucOBP8 | AFJ54049 |  | SfurOBP6 | KF732021 |
|  | AlucOBP9 | AFJ54050 |  | SfurOBP8 | KF660219 |
|  | AlucOBP10 | AFJ54051 |  | SfurOBP9 | KF732018 |
|  | AlucOBP11 | AFJ54052 |  | SfurOBP10 | KF732019 |
|  | AlucOBP12 | AFJ54053 |  | SfurOBP11 | KF732020 |
|  | AlucOBP13 | -- | ***Nilaparvata lugens*** | NlugOBP1 | ACI30679 |
|  | AlucOBP14 | -- |  | NlugOBP2 | ACI30680 |
|  | AlucOBP15 | -- |  | NlugOBP3 | ACI30681 |
|  | AlucOBP16 | -- |  | NlugOBP4 | AGZ04895 |
|  | AlucOBP17 | -- |  | NlugOBP5 | AGZ04896 |
|  | AlucOBP18 | -- |  | NlugOBP6 | AGZ04897 |
|  | AlucOBP19 | -- |  | NlugOBP7 | AGZ04898 |
|  | AlucOBP20 | -- | ***Laodelphax striatella*** | LstrOBP1 | AGZ04920 |
|  | AlucOBP21 | -- |  | LstrOBP2 | AGZ04921 |
|  | AlucOBP22 | -- |  | LstrOBP3 | AGZ04922 |
|  | AlucOBP23 | -- |  | LstrOBP4 | AGZ04923 |
|  | AlucOBP24 | -- |  | LstrOBP5 | AGZ04924 |
|  | AlucOBP25 | --- |  | LstrOBP6 | AGZ04925 |
|  | AlucOBP26 | -- |  | LstrOBP7 | AGZ04926 |
|  | AlucOBP27 | -- |  | LstrOBP8 | AGZ04927 |
|  | AlucOBP28 | -- |  | LstrOBP9 | AGZ04928 |
|  | AlucOBP29 | -- | ***Acyrthosiphon pisum*** | ApisOBP1 | NP_001153526 |
|  | AlucOBP30 | -- |  | ApisOBP2 | NP_001153528 |
|  | AlucOBP31 | -- |  | ApisOBP3 | NP_001153529 |
|  | AlucOBP32 | -- |  | ApisOBP4 | NP_001153530 |
|  | AlucOBP33 | -- |  | ApisOBP5 | NP_001153531 |
|  | AlucOBP34 | -- |  | ApisOBP6 | NP_001153532 |
|  | AlucOBP35 | -- |  | ApisOBP7 | NP_001153533 |
|  | AlucOBP36 | -- |  | ApisOBP8 | NP_001153534 |
|  | AlucOBP37 | -- |  | ApisOBP9 | NP_001153535 |
|  | AlucOBP38 | -- |  | ApisOBP10 | NP_001153525 |
| ***Adelphocoris lineolatus*** | AlinOBP1 | ACZ58027 |  | ApisOBP11 | CAX63068 |
|  | AlinOBP2 | ACZ58028 | ***Aphis craccivora*** | AcraOBP2 | CAR85658 |
|  | AlinOBP3 | ACZ58029 | ***Aphis fabae*** | AfabOBP2 | CAR85656 |
|  | AlinOBP4 | ACZ58030 |  | AfabOBP8 | CAR85657 |
|  | AlinOBP5 | ACZ58031 | ***Aphis glycines*** | AglyOBP2 | AHJ80888 |
|  | AlinOBP6 | ACZ58032 |  | AglyOBP3 | AHJ80889 |
|  | AlinOBP7 | ACZ58085 |  | AglyOBP4 | AHJ80890 |
|  | AlinOBP8 | ACZ58079 |  | AglyOBP5 | AHJ80891 |
|  | AlinOBP9 | ACZ58080 |  | AglyOBP7 | AHJ80893 |
|  | AlinOBP10 | ACZ58081 |  | AglyOBP8 | AHJ80894 |
|  | AlinOBP11 | ACZ58082 |  | AglyOBP9 | AHJ80895 |
|  | AlinOBP12 | ACZ58083 |  | AglyOBP10 | AHJ80896 |
|  | AlinOBP13 | ACZ58084 |  | AglyOBP11 | AHJ80897 |
|  | AlinOBP14 | ACZ58086 | ***Aphis gossypi*** | AgosOBP2 | AGE97632 |
| ***Lygus lineolaris*** | LlinOBP1 | AHF71028 |  | AgosOBP3 | AGE97633 |
|  | LlinOBP2 | AHF71029 |  | AgosOBP4 | AGE97634 |
|  | LlinOBP3 | AHF71030 |  | AgosOBP5 | AGE97635 |
|  | LlinOBP4 | AHF71031 |  | AgosOBP7 | AGE97637 |
|  | LlinOBP5 | AHF71032 |  | AgosOBP8 | AGE97638 |
|  | LlinOBP6 | AHF71033 |  | AgosOBP9 | AGE97639 |
|  | LlinOBP7 | AHF71034 |  | AgosOBP10 | AGE97640 |
|  | LlinOBP8 | AHF71035 | ***Brevicoryne brassicae*** | BbraOBP3 | HQ896242 |
|  | LlinOBP9 | AHF71036 | ***Drepanosiphum platanoidis*** | DplaOBP3 | HQ896238 |
|  | LlinOBP10 | AHF71037 | ***Metopolophium dirhodum*** | MdirOBP1 | CAR85638 |
|  | LlinOBP11 | AHF71038 |  | MdirOBP2 | CAR85639 |
|  | LlinOBP12 | AHF71039 |  | MdirOBP3 | CAX63256 |
|  | LlinOBP13 | AHF71040 |  | MdirOBP4 | CAR85640 |
|  | LlinOBP14 | AHF71041 |  | MdirOBP5 | CAR85641 |
|  | LlinOBP15 | AHF71042 |  | MdirOBP8 | CAR85643 |
|  | LlinOBP16a | AHF71043 | ***Megoura viciae*** | MvicOBP1 | CAR85650 |
|  | LlinOBP17 | AHF71045 |  | MvicOBP2 | CAR85651 |
|  | LlinOBP18a | AHF71046 |  | MvicOBP5 | CAR85652 |
|  | LlinOBP19 | AHF71049 |  | MvicOBP8 | CAR85653 |
|  | LlinOBP20 | AHF71050 |  | MvicOBP10 | CAX63260 |
|  | LlinOBP21 | AHF71051 | ***Myzus persicae*** | MperOBP3 | ACI30682 |
|  | LlinOBP22 | AHF71052 |  | MperOBP4 | CAR85645 |
|  | LlinOBP23 | AHF71053 |  | MperOBP7 | ACI30684 |
|  | LlinOBP24 | AHF71055 |  | MperOBP8 | ACI30683 |
|  | LlinOBP25 | AHF71056 |  | MperOBP10 | ACJ64043 |
|  | LlinOBP26 | AHF71057 | ***Nasonovia ribis-nigri*** | NribOBP2 | CAR85654 |
|  | LlinOBP27 | AHF71058 |  | NribOBP3 | CAX63257 |
|  | LlinOBP28 | AHF71059 |  | NribOBP7 | CAX63259 |
|  | LlinOBP29 | AHF71060 |  | NribOBP8 | CAR85655 |
|  | LlinOBP30 | AHF71061 | ***Pterocomma salicis*** | PsalOBP1 | CAR85660 |
|  | LlinOBP31 | AHF71062 |  | PsalOBP2 | CAR85661 |
|  | LlinOBP32 | AHF71063 |  | PsalOBP4 | CAR85662 |
| ***Adelphocoris suturalis*** | AsutOBP6 | AHJ81241 |  | PsalOBP9 | CAR85663 |
|  | AsutOBP7 | AHJ81239 |  | PsalOBP10 | CAX63261 |
|  | AsutOBP8 | AHJ81242 | ***Rhopalosiphum padi*** | RpadOBP2 | CAX63253 |
|  | AsutOBP10 | AHJ81240 |  | RpadOBP5 | CAX63254 |
|  | AsutOBP11 | AHJ81243 |  | RpadOBP10 | CAX63255 |
|  | AsutOBP12 | AHJ81244 | ***Sitobion avenae*** | SaveOBP2 | JN165749 |
| ***Rhodnius prolixus*** | RproOBP2 | CAX63262 |  | SaveOBP3 | HQ896243 |
|  | RproOBP4 | CAX63263 |  | SaveOBP4 | JN165751 |
|  | RproOBP5 | CAX63264 |  | SaveOBP7 | GQ847859 |
|  | RproOBP6 | CAX63265 |  | SaveOBP8 | GQ888708 |
| ***Euschistus heros*** | EherOBP1 | ADJ18275 |  | SaveOBP9 | GQ847860 |
|  | EherOBP2 | ADO24165 |  | SaveOBP10 | FN293384 |

“--” means that genes proteins Acc. Number have not obtained from NCBI.
