# Supplementary material for: Molecular Characterization and Expression Profiling of Odorant-Binding Proteins in Apolygus lucorum
Source: PLoS One. 2015 Oct 14;10(10):e0140562. doi: 10.1371/journal.pone.0140562 (PMC4605488; doi:10.1371/journal.pone.0140562)
Supplement: S4 Table — (DOCX) [file pone.0140562.s004.docx]

**Supplementary materials**

**S4 Table. Primers used for qRT-PCR**

| **Genes** | **Forward (5'-3')** | **Reverse (5'-3')** |
| --- | --- | --- |
| AlucOBP13 | AAGCCGAACTACCATCAGACATTA | CGTGTGTTTGGCTATCAGGTCTTC |
| AlucOBP14 | TTTGAAGGCAACGCTAAAAGACT | CAGGGTTCTCGTGAAGGATGTT |
| AlucOBP15 | AAGAAGACTGTCATGCTGGATGT | CATTCACTCTTGCCTTTGGTATCTC |
| AlucOBP16 | TCGGCTACTTGACAGGTGGAC | CTTTCTTCCCGTTTGTAACACTCTG |
| AlucOBP17 | GAGAGAAATGGCTCAGGGACTT | CGAAATGACTCCGAGGTTGC |
| AlucOBP18 | CTCCGCCCTTCTTGTGGCTTAT | GCAAGAAGCACCTTTCAGCAT |
| AlucOBP19 | GCCAAAATCGCAGAAATCAAA | GTCAGCGGGGTCCTCGTAT |
| AlucOBP20 | GCCGATGAACCTGCTGAGT | TTCACCGTAACCGCTTCAAGTT |
| AlucOBP21 | CCCGTCACCGATGAGGAAAT | CGTGCTCAGGTTCGTCTTTGTA |
| AlucOBP22 | TCAAGGACCACCCTGAGAAAC | GCCAAGTCGCATTCGTTGT |
| AlucOBP23 | TTCACTGTAGCCTTGTCTT | CTTCTTCAAAGCACTTATGGA |
| AlucOBP24 | TCCGTTCGGTTGGGATGAT | CAAGCACCTTTCCTCCTGATTTT |
| AlucOBP25 | ACAAGCCCGAAGTTATCAAGAAGT | TGGTCCAACTCTCCGTCTGA |
| AlucOBP26 | AAAAGTCAAAGTTCAGTGCCAGC | ATCCCGCCAGTCTTCGTGT |
| AlucOBP27 | CCTCGTCGCTTTGTTGGTATT | CTGTCGAATTTTTACACTGTTTTAGA |
| AlucOBP28 | AGCGAAGAAAGTGATGACCAAA | TCGGGAATGTAGTCTAAAAACGG |
| AlucOBP29 | GAGACCACCTCAATGTTGCGAT | CCTGTTGCCGATTTGCCTG |
| AlucOBP30 | GCATCACCACGACAGAGAC | GCACCAGGCTCATTGAACTT |
| AlucOBP31 | TTATGGACTGAGAGCACGGACT | GATGATGACCGATTGGCACCT |
| AlucOBP32 | CAAATAAACACGACTGTATGACCGAG | GCATTCGGTTGAACTGAACTTTTTT |
| AlucOBP33 | CTAATACTCTTCCGACTCTTGGGATACA | ACTCACTTCCTTGGGCAACTCC |
| AlucOBP34 | GTCAAAACAACCATCCAAAGCAA | ATCGGGTCCTATCTGAGTTACGC |
| AlucOBP35 | GAGCCCGAATGATAAAGAACGA | TTCAGACGGGTTCTCACCATTAA |
